# The Purchasing Power of a Sovereign

**Published:** 1914-03-21

**Authors:** 


### THE PURCHASING POWER OF A SOVEREIGN.

It would be an imense gain to women as a class, and especially to heads of households, with the responsibility and heavy demands which attach to the position of the wife of a practitioner, did they appreciate at its true value the purchasing power of a sovereign and the causes which create notable fluctuations in its value from time to time.

For instance, in the years preceding 1906 the purchasing power to the worker in this country was considerably higher than in the years that have followed. Professor Marshall has clearly indicated that a continuous depression in prices, a depression in interest and a depression in profits, is consistent with a condition of prosperity to the worker, although the employer in such circumstances gets less. When prices rise, interest increases and profits increase with them, as has been the case since 1896, owing partly, it is true, to the frequency of labour disputes in England, Germany, France, and America. The cost of living has gone up and the purchasing power of the sovereign has diminished markedly, especially in the case of the less wealthy workers. It has been stated recently by those who have advocated a Trades Union of Wives that the Board of Trade figures show that the retail price in London of twenty-three of the principal foodstuffs, including bread, meat, bacon, butter, eggs, and the majority of foods bought by the workers, has advanced by practically 18 per cent. during 1896 to 1910. During the last seventeen years it is declared that the purchasing power of a sovereign has fallen to the extent of 5s., or by 25 per cent. These marked differences mainly apply to those who buy everything retail and who follow the habit, dear to many women, to act individually—that is, to buy everything on their own. We have reason to feel confident that, *excluding food of all kinds*, a co-operation of doctors' wives may restore to all who join this organisation a return of from two-fifths to four-fifths of the fall in the earning power of a sovereign just illustrated.

